# Claudin-18 expression under hyperoxia in neonatal lungs of bronchopulmonary dysplasia model rats

**DOI:** 10.3389/fped.2022.916716

**Published:** 2022-10-10

**Authors:** Jingye Zuo, Yajie Tong, Yuting Yang, Yirui Wang, Dongmei Yue

**Affiliations:** Department of Pediatrics, Shengjing Hospital of China Medical University, Shenyang, China

**Keywords:** bronchopulmonary dysplasia, claudin-18, canonical WNT pathway, AEC transdifferentiation, claudin-4

## Abstract

**Background:**

Bronchopulmonary dysplasia (BPD) is characterized by impaired alveolar and microvascular development. Claudin-18 is the only known lung-specific tight junction protein affecting the development and transdifferentiation of alveolar epithelium.

**Objective:**

We aimed to explore the changes in the expression of claudin-18, podoplanin, SFTPC, and the canonical WNT pathway, in a rat model of hyperoxia-induced BPD, and to verify the regulatory relationship between claudin-18 and the canonical WNT pathway by cell experiments.

**Methods:**

A neonatal rat and cell model of BPD was established by exposing to hyperoxia (85%). Hematoxylin and eosin (HE) staining was used to confirm the establishment of the BPD model. The mRNA levels were assessed using quantitative real-time polymerase chain reaction(qRT-PCR). Protein expression levels were determined using western blotting, immunohistochemical staining, and immunofluorescence.

**Results:**

As confirmed by HE staining, the neonatal rat model of BPD was successfully established. Compared to that in the control group, claudin-18 and claudin-4 expression decreased in the hyperoxia group. Expression of β-catenin in the WNT signaling pathway decreased, whereas that of p-GSK-3β increased. Expression of the AEC II marker SFTPC initially decreased and then increased, whereas that of the AEC I marker podoplanin increased on day 14 (*P* < 0.05). Similarly, claudin-18, claudin-4, SFTPC and β-catenin were decreased but podoplanin was increased when AEC line RLE-6TN exposed to 85% hyperoxia. And the expression of SFTPC was increased, the podoplanin was decreased, and the WNT pathway was upregulated when claudin-18 was overexpressed.

**Conclusions:**

Claudin-18 downregulation during hyperoxia might affect lung development and maturation, thereby resulting in hyperoxia-induced BPD. Additionally, claudin-18 is associated with the canonical WNT pathway and AECs transdifferentiation.

## Introduction

Bronchopulmonary dysplasia (BPD) is one of the most common chronic respiratory diseases in preterm infants. Lung injury caused by inflammation is the key factor in BPD (1, 2), the typical pathological feature of which is the interruption of alveolar and capillary development. Pulmonary edema is the main manifestation of BPD in the early stage (3). Alveolar epithelial barrier is a sealed interface between adjacent epithelial cells, whose damage could lead to pulmonary edema, and its function depends on tight junctions—a type of transmembrane protein complex.

The claudin family of membrane proteins plays a central role in the structure and function of tight junctions (4). In humans, claudin-1, 3–5, 7, 8, 10, 12, 18, and 23 are most abundantly expressed (5). Studies in rats demonstrate that alveolar epithelial cells (AECs) I and II primarily express claudin-3, 4, and 18 (6). However, only claudin-18.1, one of the isomers of claudin-18, is known to be specifically expressed in lung tissues (7), indicating its lung-specific functions.

The canonical WNT pathway has been reported to be extensively involved in lung cell proliferation, migration, invasion, and apoptosis (8–10), playing a key role in the patterning of early lung organogenesis (11). WNT signaling in epithelial and mesenchymal cells peaks at the onset of the tubular phase, declines sharply in the mid-tubular phase, and remains low during the cystic and alveolar phases (12). Wnt3a can regulate the proliferation and differentiation of AECII to AECI in hyperoxia-induced BPD (13).

Many studies have proven that the claudin family is closely related to the WNT/β-catenin pathway. Regulation of claudin-18 is involved in the JAM axis and closely related to the WNT/β-catenin pathway (14). JAM-A is a putative inhibitor of glycogen synthase kinase-3β (GSK-3β) (15). And mice with low expression of JAM-A have been reported to show increased sensitivity to lung injury (16). Therefore, JAM-A might have a protective effect against acute lung injury by stimulating alveolar epithelial tight junctions. The purinergic receptor P2X7, representing an ATP-gated ionotropic receptor, affects the WNT/β-catenin signaling pathway in AECs under conditions of injury (17). Expression of claudin-18 in the lungs of P2X7-/- mice has been reported to be increased, and the expression of GSK-3β protein and its inactive form in AECs also increased, suggesting that claudin-18 is negatively correlated with P2X7 receptor expression and positively correlated with GSK-3β expression related (18). Precisely coordinated activation of the WNT/β-catenin signaling pathway is critical for normal lung development. Aberrantly activated β-catenin patterns have been reported in human BPD tissues and in hyperoxia models of BPD. Increased plasma wnt5a in hyperoxia-induced lung cystic hyperplasia could promote alveolarization and septal thickening in BPD patients (19). However, the mechanism by which the WNT pathway specifically regulates claudin-18 expression still remains unclear.

**Figure 1:**
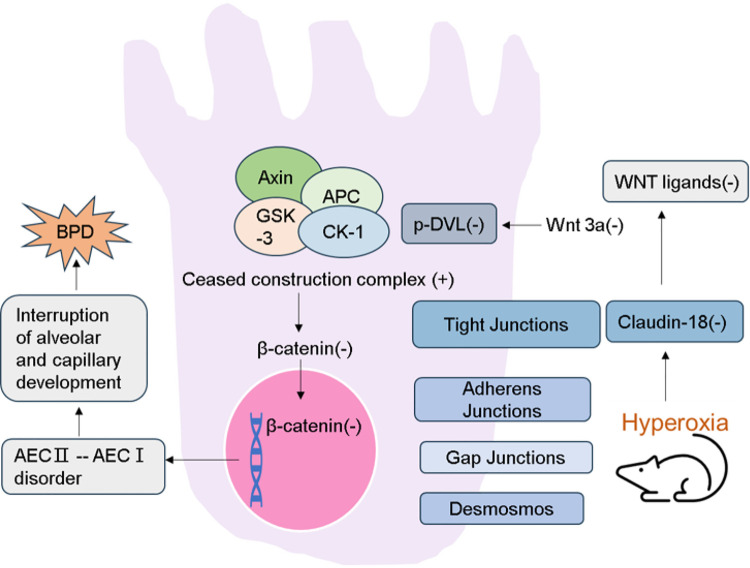
Schematic demonstration of the current regulatory model. Expression of Claudin-18 is decreased in lung tissue of rat model of hyperoxia-induced BPD. Then the WNT classical pathway is inhibited. And the destruction ability of β-catenin degradation complex was increased. As a result, the accumulation of β-catenin in the cytoplasm is reduced and its entry into the nucleus is reduced. Activating WNT pathway promotes transdifferentiation of AECs, which leads to the interruption of alveolar and capillary development.

AECs are injured when exposed to hyperoxia, which might lead to the pathogenesis of BPD, which is regulated by many factors, such as connective tissue growth factor (20), Nrf2 (21), and silencing information regulator 2 related enzyme 1 (22) and so on. Claudin-18 is an important component of pulmonary tight junction proteins involved in permeability regulation and barrier function, and it has also been shown to be involved in the process of alveolar development (23). Claudin-4 is a type of transmembrane protein that promotes fluid-clearance function to maintain function of lung epithelial barrier (24, 25). We hypothesized that claudin-18 downregulation during hyperoxia could affect lung development and contribute to hyperoxia-induced BPD. And claudin-18 is associated with the canonical WNT pathway and AECs transdifferentiation (Figure 1). In this study, we assessed the changes in claudin-18, claudin-4, SFTPC, podoplanin, GSK-3β, p-GSK-3β, and β-catenin expression levels in the lungs of neonatal rat model of hyperoxia-induced BPD compared to that in control rats. Additionally, we aimed to study the development of alveoli, to further illustrate the role of claudin-18 in the pathophysiological process of hyperoxia-induced BPD.

## Materials and methods

### Experimental subjects and groups

All animal experiments were performed in accordance with the National Institutes of Health guidelines and protocols approved by the Institutional Animal Care and Use Committee (approval no. 2020PS574K). Forty Sprague–Dawley rats (10-week-old; Changsheng Co., Ltd. Shenyang, China) were mated at a male to female ratio of 1:4. Female rats were separated after conception. Within 12 h of the pregnant female rats naturally giving birth, neonatal rats were randomly divided into two groups namely Con (21% O2, *n* = 50) and BPD (80%–85% O2, *n* = 50). The newborn rats, along with their mother rats were exposed to normoxia or hyperoxia until postnatal day 14. The lactating mother rats were switched between normoxia and hyperoxia every 24 h to avoid oxygen poisoning. All animals had a 12-h light/dark cycle, with free drinking water and standard diet (23).

### Tissue harvest

Ten neonatal rats were randomly selected on postnatal days 1, 3, 7, 10, and 14 (Supplementary Figure S1) for harvesting their lungs. The thoracic cavity was opened after euthanasia by isoflurane (2%) inhalation, and the right ventricle was perfused. When lungs became white, they were collected aseptically. For preparing paraffin specimens for hematoxylin and eosin (HE) staining, immunohistochemistry (IHC), and immunofluorescence (IF), the right lower lobe was fixed in 4% paraformaldehyde without dissection, dehydrated using graded alcohol, and rendered transparent using xylene. The remaining lobes were quick-frozen in liquid nitrogen and stored at −80 °C for subsequent western blot analysis and qPCR.

### Determination of wet and dry weights

To determine the wet and dry weights, the upper right lobe was collected and weighed as wet weight. It was then placed in an oven at 80 °C for 24 h, and then weighed again. This step was repeated until the weight of the lung lobe changed no further, and dry weight was obtained. The psychrometric ratio, given by = (wet weight-dry weight)/dry weight, was calculated to assess the degree of pulmonary edema.

### Hematoxylin and eosin (He) staining and radical alveolar count (RAC)

Paraffin blocks of lung tissues were sliced into 2.5-*μ*m thick sections for HE staining. Radical alveolar count (RAC; Wang et al. 2019) was determined as follows: Based on the HE-stained specimens, a straight line was drawn directly between the centre of the respiratory bronchioles and the nearest fibrous septum, and the number of alveoli through which the line passed were counted. RAC was determined using a light microscope (magnification, 100×) by counting each slice thrice and averaging the obtained values.

### Quantitative real-time PCR

Total RNA was extracted from lung tissues using the RNAiso Plus (Code No9108, TaKaRa Bio, Japan), and concentration and purity of the isolated RNA were measured using a NanoDrop spectrophotometer (Thermo Fisher Scientific, MA, USA). For removing genomic DNA contamination and reverse-transcribing the obtained RNA, the PrimeScriptTM RT reagent kit with gDNA Eraser kit (Code No. RRo47A, Takara Bio, Japan) was used. PCR amplification was conducted using qPCR premix (Code No. RR820A, Takara Bio, Japan). Primers were designed by Sangon Biotech; the sequences and other details are shown as follows: Rat ACTB Endogenous Reference Genes Primers (Order NO. B661202, Sangon Biotech); claudin-18 PCR primers: GCTCTGTTTGTGGGCT GGATCG (forward) and GAAGTTGCGGTCATCAG GA GTCAG (reverse); claudin-4 PCR primers: TCATCGGCAGCAACATCGTCAC(forward) and GCC AGCATCG AGTCGTACATCTTG (reverse); SFTPC PCR primers: CCCAGGAGCCAGTTTCGC ATTC(forward) and GACGACAAGGACTACCACCACAAC (reverse); podoplanin PCR primers: C CTCCGACCACGATCACAAAGAAC (forward) and CACCGCCT GCGTTATCTCTATTGG (reverse); β-catenin PCR primers: TACCGCTGGGACCC TACACAAC(forward) and GCG TGGTGATGGCGTAGAACAG (reverse).

The cycle threshold (Ct) value was considered acceptable for analysis. ACTB was used as the reference gene, and the 2^−ΔΔCt^ method was used to evaluate the relative changes in levels of each gene. All data shown represent the average results from three replicates.

### Western blot analysis

Total protein was extracted from the lung tissue, and its concentration was determined using an enhanced BCA kit (Beyotime Biotechnology). Thereafter, 30 *µ*g of the protein sample was subjected to 10% SDS-polyacrylamide gel electrophoresis, followed by transfer of the separated proteins to a polyvinylidene fluoride membrane (ISEQ00010, Millipore, USA) (26). After blocking with 5% skim milk at 37 °C for 2 h, the membrane was incubated with anti-claudin-18 (1:1,000, Abcam, USA), anti-claudin-4 (1:1,000, Invitrogen, USA), anti-SFTPC (1:1,000, Proteintech, China), anti-podoplanin (1:1,000, Santa Cruz, USA), anti-GSK-3β (1:1,000, CST,USA), anti-p-GSK-3β (1:1,000, CST, USA), and anti-β-catenin (1: 1,000, CST, USA) antibodies overnight at 4 °C. The next day, membranes were incubated with the corresponding horseradish peroxide-conjugated secondary antibody (S0001, S0002, Affinity) at 37 °C for 2 h. Amersham Imager 600 (GE Healthcare Life Sciences) was used to detect chemiluminescence signals, and the Image J 1.8.0 was used to calculate the gray value. The experiment was repeated at least thrice.

### Immunohistochemical staining

Paraffin sections were deparaffinized, incubated with Tris-EDTA antigen retrieval solution, and blocked with anti-claudin-18 (1:200), anti-claudin-4 (1:100,), anti-SFTPC (1:100), anti-podoplanin (1:200), and anti-β-catenin (1:100) antibodies at 4 °C overnight. Images were acquired and analysed using Image Pro Plus 6.0 to assess protein expression.

### Immunofluorescence staining

Paraffin sections (2.5-µm thick) were deparaffinized, washed with PBS, heat-repaired, and blocked with 3‰ Triton-X in 10% goat serum at room temperature for 1 h. The specimens were then incubated with anti-claudin-18 (1:200), anti-β-catenin (1:100), or anti-podoplanin-antibodies (1:100) overnight at 4 °C. The next day, sections were incubated with Alexa Fluor 488- and Alexa Fluor 594-conjugated secondary antibodies (1:400, Abcam) for 4 h at37 °C. Sections were subjected to DAPI staining for 5 min, following which they were mounted with glycerol and visualized under a full spectrum laser confocal microscope (C1Si, Nikon, Japan).

### Cell culture and transfection

The RLE-6TN cell line was purchased from Bena Culture Collection and cultured in RPMI 1,640 medium (Gibco) with 10% fetal bovine serum (Clark). The control group was maintained in a humidified incubator with 21% O2 while the BPD group was kept at 37 °C with 85% O2, and cells were collected after 24 h. The cells were transfected with negative control (NC) plasmid to form the P-NC group, and with claudin18 overexpression plasmid synthesized by Hanheng Co., Ltd. to generate the claudin18 group. Transfections were performed using Lipo 3,000 (Invitrogen) according to the manufacturer's instruction.

### Statistical analysis

The data obtained in the experiment were all analysed using Prism 8.0. A t-test was performed while assessing two groups, whereas one-way analysis of variance (ANOVA) was performed for comparing multiple groups, and repeated measures ANOVA was employed for comparing two groups at different time points. Statistical significance was set at 0.05.

## Results

### Pulmonary edema and alveolar dysfunction observed in the neonatal rat model of hyperoxia-induced BPD

**Figure 2:**
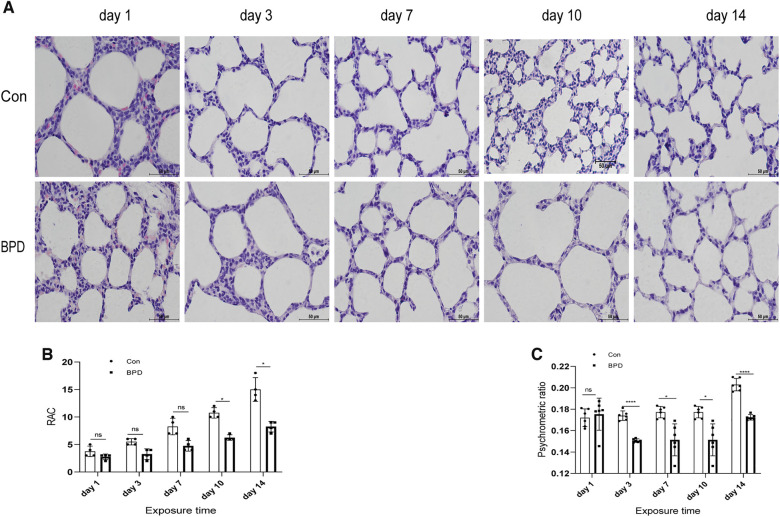
Appearance and histomorphological changes of neonatal rat lung tissues. (**A**) HE staining of lung tissue of neonatal rats under air and hyperoxia (magnification ×200). (**B**) RAC analysis of neonatal rats under air and hyperoxia. (**C**) Psychrometric ratio analysis of neonatal rats under air and hyperoxia. Repeated measures ANOVA *P < 0.05, **P < 0.01, ****P < 0.0001 vs. Con, *n* = 3.

The survival rate of neonatal rats was 100% in both control group and BPD group. Lung tissues of neonatal rats in the hyperoxia group showed evident edema and immature alveolar development. Histopathological results showed that, with increase in age, alveolar cavity of the air group rats increased in number and decreased in size; the alveolar compartments increased in number and gradually became thinner. In the BPD group, alveolar development gradually became disordered, volume of the alveolar cavity became larger with evident alveolar fusion, interstitial cell proliferation, and simultaneous inflammatory cell infiltration (Figures 2A,B). Psychrometric ratio analysis suggested that the degree of lung tissue edema in neonatal rats of the BPD group was significantly higher than that in the control group (Figure 2C).

### Down-regulation of claduin-18 and claudin-4 in the rat model of hyperoxia-induced BPD

**Figure 3:**
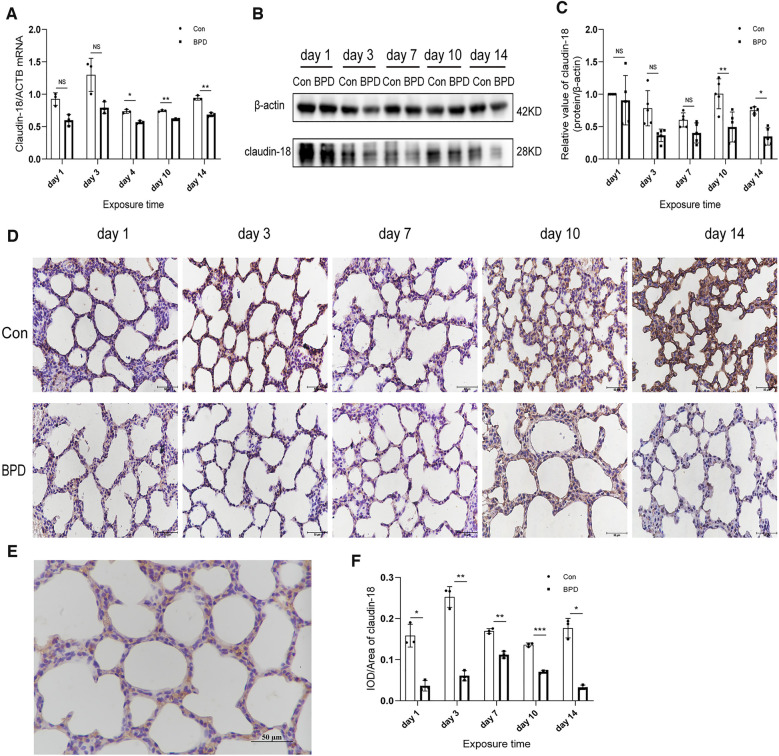
Decreased claudin-18 levels in the lung tissue of neonatal rats under hyperoxia. (**A**) Relative mRNA expression of claudin-18 in the lung tissue of neonatal rats under air and hyperoxia. (**B,C**) Protein expression of claudin-18 in the lung tissue of neonatal rats under air and hyperoxia. (**D**) IHC showing the expression of claudin-18 in the lung tissue of neonatal rats under air and hyperoxia at 5 time points (magnification, 400×). (**E**) Location of claudin-18 in the lung tissues. (**F**) Semi-quantitative analysis of claudin-18 expression in the lung tissues based on IHC. Repeated measures ANOVA, *P < 0.05 vs. Con, qPCR and IHC *n* = 3, western blot *n* = 5.

**Figure 4:**
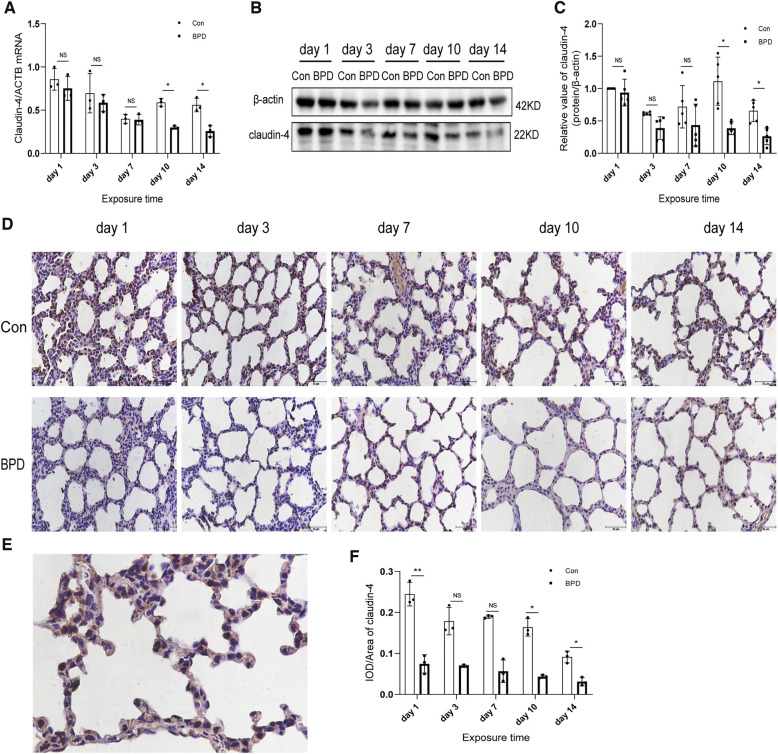
Decreased claudin-4 levels in the lung tissue of neonatal rats under hyperoxia. (**A**) Relative mRNA expression of claudin-4 in the lung tissue of neonatal rats under air and hyperoxia. (**B,C**) Protein expression of claudin-4 in the lung tissue of neonatal rats under air and hyperoxia. (**D**) IHC showing the expression of claudin-4 in the lung tissue of neonatal rats under air and hyperoxia at 5 time points (magnification, 400×). (**E**) Location of claudin-4 in the lung tissues. (**F**) Semi-quantitative analysis of claudin-4 expression in the lung tissues based on IHC. Repeated measures ANOVA, *P < 0.05 vs. Con, qPCR and IHC *n* = 3, western blot *n* = 5.

To determine the role of claudin-18 in BPD model induced by hyperoxia, we used different methods to detect the expression of claudin-18 (Figure 3) and claudin-4 (Figure 4) in neonatal lung tissues. QPCR results showed that claudin-18 transcription in the neonatal lung of the control group to be significantly higher than that in the BPD group (*P* < 0.05) (Figure 3A). Western blot experiments showed that, compared to the air group, claudin-18 expression was significantly reduced, to varying degrees, under hyperoxia (*P* < 0.05) (Figures 3B,C). IHC results showed claudin-18to mainly be expressed in the cytoplasm of AECs (Figures 3D,E). Semi-quantitative analysis showed claudin-18 expression in lung tissues of the air group to be significantly higher than that in the BPD group (*P* < 0.05) (Figure 3F). The experimental results are perfectly uniform, seemingly proving that hyperoxia affects claudin-18 expression in pulmonary tissues.

We next used the three methods mentioned above to detect claudin-4, another important member of the claudin family. QPCR results showed that claudin-4 transcription in the air group was significantly higher than that in the hyperoxia group on days 10 and 14; however, no difference in claudin-4 expression was noted between the air and hyperoxia groups on postnatal days 1, 3, and 7 (*P* > 0.05) (Figure 4A). Western blot results showed no significant difference in claudin-4 expression between these two groups on postnatal days 1 and 7 (*P* > 0.05). However, claudin-4 expression in the control group was significantly higher than that in BPD group on postnatal days 10and 14 (*P* < 0.05) (Figures 4B,C). IHC showed claudin-4 to be primarily expressed in the cytoplasm of AECs (Figures 4D,E); claudin-4 expression in the hyperoxia group was significantly lower than that in the air group (*P* < 0.05) (Figure 4F). Expression of claudin-18 and claudin-4 was decreased in the rat model of hyperoxia-induced BPD.

### Podoplanin and SFTPC expression in the rat model of hyperoxia-induced BPD

**Figure 5:**
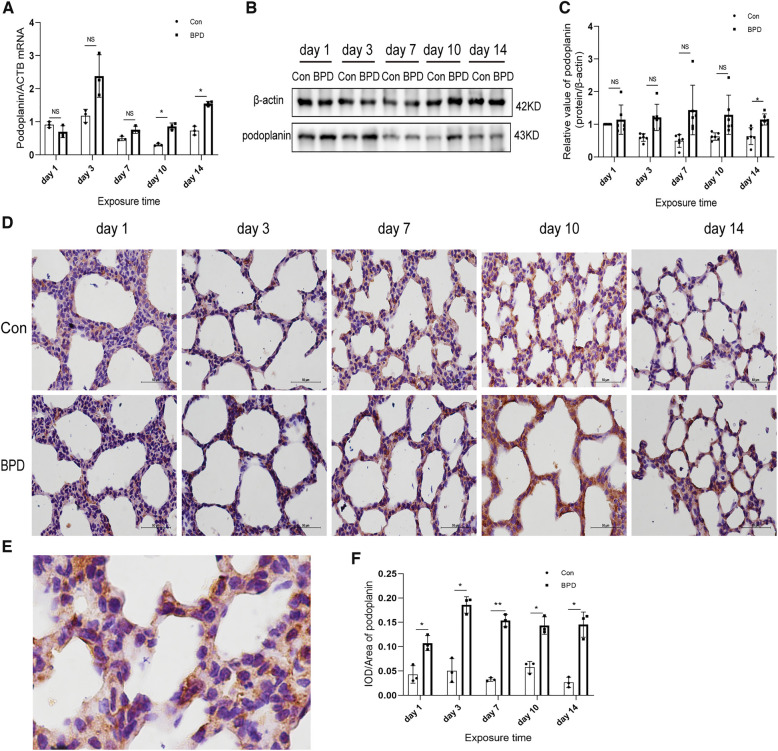
Increased podoplanin levels in the lung tissue of neonatal rats under hyperoxia. (**A**) Relative mRNA expression of podoplanin in the lung tissue of neonatal rats under air and hyperoxia. (**B,C**) Protein expression of podoplanin in the lung tissue of neonatal rats under air and hyperoxia. (**D**) IHC showing the expression of podoplanin in the lung tissue of neonatal rats under air and hyperoxia at 5 time points (magnification, 400×). (**E**) Location of podoplanin in the lung tissues. (**F**) Semi-quantitative analysis of podoplanin expression in the lung tissues based on IHC. Repeated measures ANOVA, *P < 0.05, **P < 0.01 vs. Con, qPCR and IHC *n* = 3, western blot *n* = 6.

**Figure 6:**
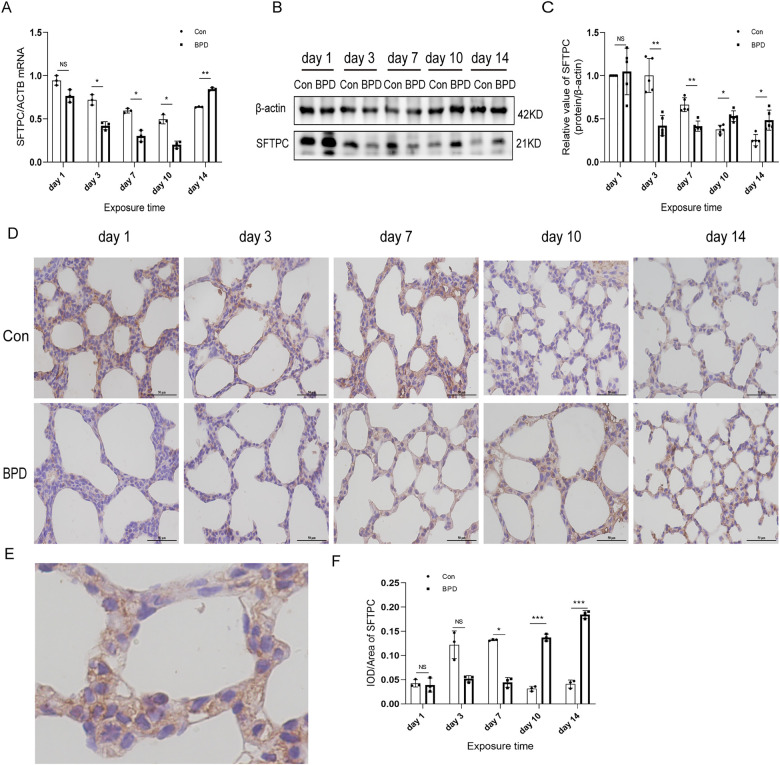
SFTPC levels in the lung tissue of neonatal rats under hyperoxia. (**A**) Relative mRNA expression of SFTPC in the lung tissue of neonatal rats under air and hyperoxia. (**B,C**) Protein expression of SFTPC in the lung tissue of neonatal rats under air and hyperoxia. (**D**) IHC showing the expression of SFTPC in the lung tissue of neonatal rats under air and hyperoxia at 5 time points (magnification, 400×). (**E**) The Location of SFTPC in the lung tissues. (**F**) Semi-quantitative analysis of SFTPC expression in the lung tissues based on IHC. Repeated measures ANOVA, *P < 0.05, **P < 0.01, ***P < 0.001 vs. Con, qPCR and IHC *n* = 3, western blot *n* = 5.

We tested the expression of marker proteins AEC I-podoplanin and AEC II-SFTPC to confirm the transdifferentiation of AECs under hyperoxia (Figures 5, 6). QPCR results showed that podoplanin in the hyperoxia group to be significantly higher than that in the air group on days 10 and 14 (Figure 5A). Western blot results showed podoplanin expression to only be increased significantly on postnatal day 14, with no difference in the expression levels between the hyperoxia and air groups on days 1, 3, 7, and 10 (Figures 5B,C). Similar to the qPCR results, IHC showed that under hyperoxia, podoplanin expression was significantly increased (Figures 5D–F).

The SFTPC transcription in neonatal lungs of the hyperoxia group was significantly lower than that in the air group on postnatal days 3, 7, and 10 while being significantly higher on day 14 (Figure 6A). Western blot results suggested that SFTPC expression in the BPD group was significantly lower than that in the control group on postnatal days 3 and 7, whereas it was significantly higher in the BPD group on postnatal days 10 and 14 (Figures 6B,C). IHC suggested that SFTPC was expressed in both nucleus and cytoplasm (Figures 6D,E). The semi-quantitative analysis results were consistent with the western blot results (Figure 6F).

### Inhibition of the canonical WNT pathway in the rat model of hyperoxia-induced BPD

**Figure 7:**
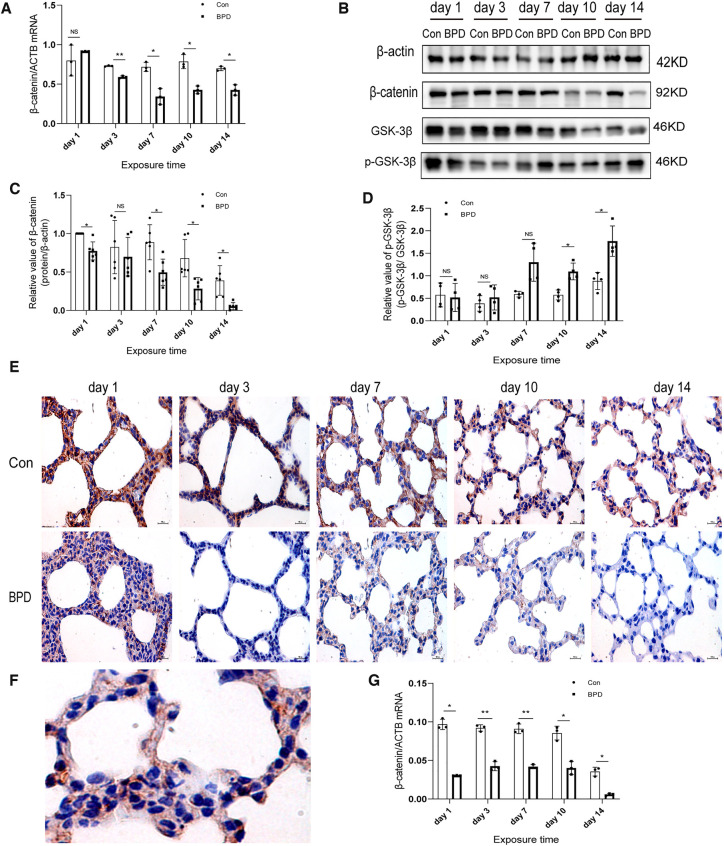
Downregulation of WNT pathway in the lung tissue of neonatal rats under hyperoxia. (**A**) Relative mRNA expression of β-catenin in the lung tissue of neonatal rats under air and hyperoxia. (**B–D**) Protein expression of β-catenin, GSK-3β, and p- GSK-3β in the lung tissue of neonatal rats under air and hyperoxia. (**E**) IHC showing the expression of β-catenin in the lung tissue of neonatal rats under air and hyperoxia at 5 time points (magnification, 400×). (**F**) The lLocation of β-catenin in the lung tissues. (**G**) Semi-quantitative analysis of β-catenin expression in the lung tissues based on IHC. Repeated measures ANOVA, *P < 0.05, **P < 0.01 vs. Con, qPCR and IHC *n* = 3, western blot *n* = 6.

We found that the WNT pathway was downregulated under hyperoxia (Figure 7). Moreover, the mRNA expression of β-catenin in the hyperoxia group was found to be significantly lower than that in the air group (Figure 7A). Western blot results showed no significant difference in β-catenin expression between the two groups on postnatal day 3; however, the expression in the hyperoxia group was significantly lower than that in the air group on postnatal days 1, 7, 10, and 14and β-catenin level was dramatically reduced on day 14 (Figures 7B,C). Further, the relative protein expression of p-GSK-3β significantly increased on days 10 and 14 (Figure 7D). IHC showed β-catenin to be expressed in both the nucleus and the cytoplasm (Figures 7E,F). Under hyperoxia, β-catenin expression was significantly reduced (Figure 7G).

### Correlation between claudin-18 and β-catenin expression in neonatal lungs

**Figure 8:**
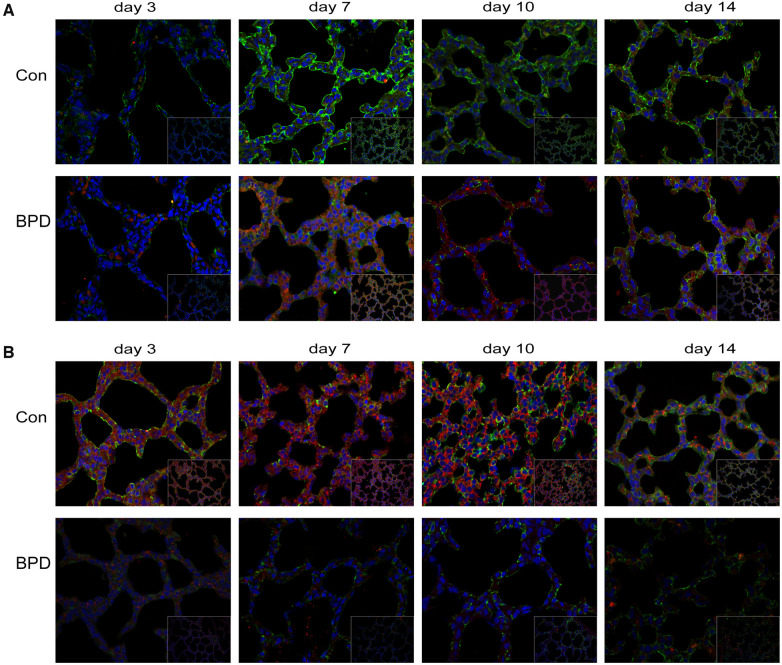
Co-expression of claudin-18 and podoplanin/β-catenin. (**A**) Claudin-18 expression in AEC I. Green fluorescence represents claudin-18, red fluorescence represents podoplanin, and orange fluorescence represents the co-expression of claudin-18 and podoplanin. (**B**) Co-expression of claudin-18 and β-catenin in neonatal lung tissues. Green fluorescence represents claudin-18, red fluorescence represents β-catenin, and orange fluorescence represents the co-expression of both proteins. *n* = 3.

Double IF staining was performed to locate claudin-18 and podoplanin in pulmonary tissues (Figure 8A). Claudin-18 was mainly expressed in the cytoplasm along the cell membrane, while podoplanin was mainly expressed within the cytoplasm. At the same time point, green fluorescence intensity of claudin-18 was higher in the Con group than that in the BPD group. As red fluorescence intensity of podoplanin was lower in the Con group, the overall colour was greener. Whereas fluorescence of the BPD group was reddish.

To observe the effect of hyperoxia on claudin-18 and β-catenin expression, we performed double IF staining of claudin-18 and β-catenin (Figure 8B). As seen earlier, claudin-18 was mainly expressed along the edge of the cytoplasmic cell membrane, whereas β-catenin was diffusely expressed in the cytoplasm. Fluorescence intensity of both was greatly reduced under hyperoxia. The red fluorescence representing by β-catenin underwent greater reduction, and fluorescence of the air group was, therefore, orange, whereas that of BPD group was green.

We conducted Pearson's correlation analysis on claudin-18 and β-catenin expression based on the western blot results and found a significant positive correlation between the two (*r* = 0.286, *P* < 0.05) (Supplementary Figure S2).

### Influence of claudin-18 overexpression on the WNT pathway and AECs transdifferentiation

**Figure 9:**
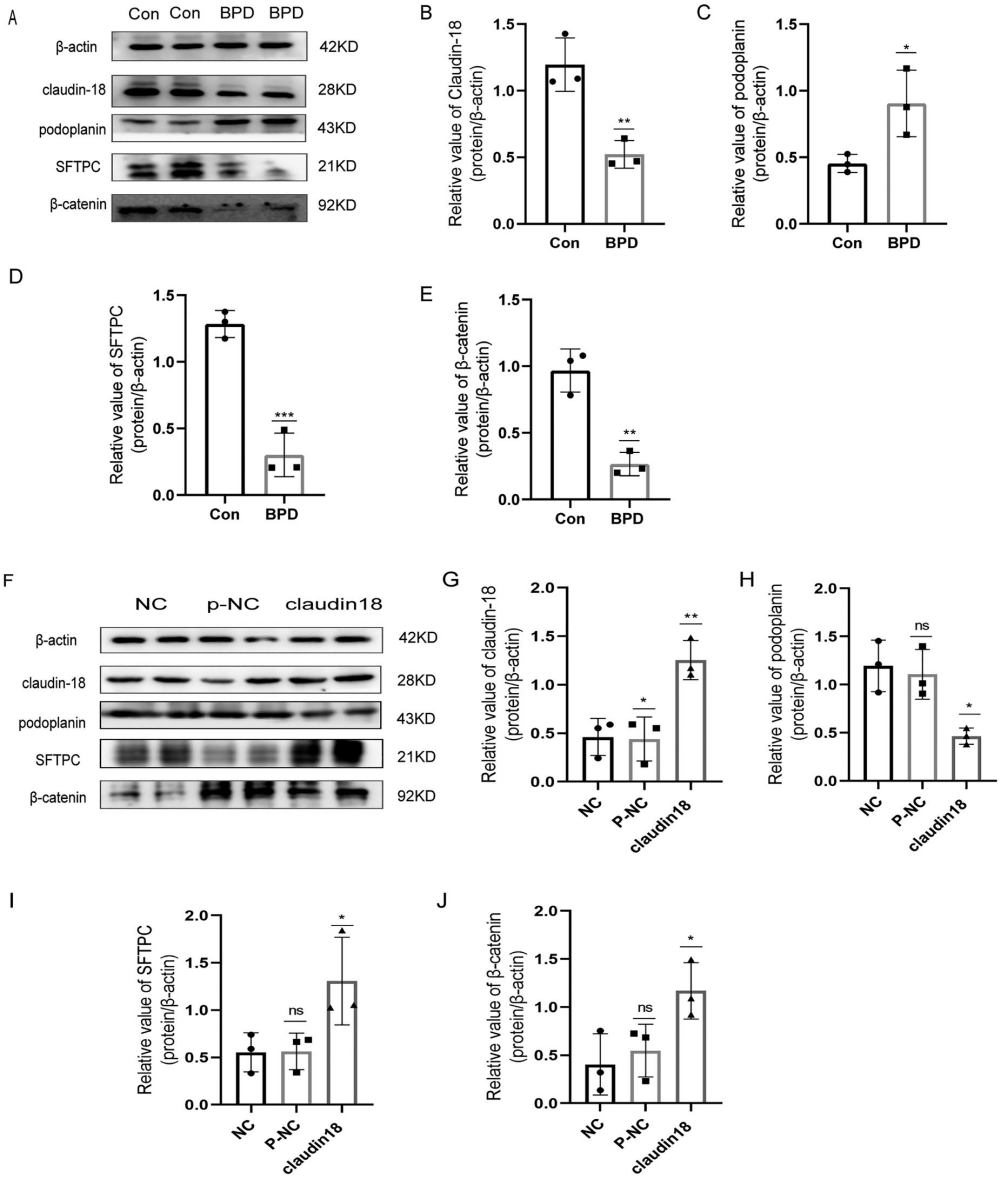
Effects of hyperoxia and claudin-18 overexpression on WNT pathway and AECs transdifferentiation. (**A–E**) Protein levels of podoplanin, SFTPC, and β-catenin upon hyperoxia. T-test analysis, *P < 0.05, **P < 0.01, ***P < 0.001 vs. Con, *n* = 3. (**F–J**) Protein levels of podoplanin, SFTPC, and β-catenin after overexpression plasmid transfection. One-way ANOVA, *P < 0.05, **P < 0.01 vs. Con, *n* = 3.

To verify the results of animal experiments, we established a BPD cell model. Our experimental results showed the expression of claudin-18, SFTPC and β-catenin to decrease and the that of podoplanin to increase under hyperoxia, which was consistent with the results of animal experiments (Figures 9A–E).

Next, we constructed a claudin18 overexpression plasmid to explore the effect of claudin18 on the canonical WNT pathway and AECs transdifferentiation (Figures 9F–J). Overexpression of claudin-18 increased the expression of SFTPC and decreased that of podoplanin, suggesting the transdifferentiation of AECs to have weakened. At the same time, the expression of β-catenin was increased, which suggested the WNT pathway to be activated.

## Discussion

In this study, we aimed to determine the role of claudin-18 in the pathophysiology of hyperoxia-induced BPD using a suitable neonatal rat model. Claudin-18 was found to be mainly distributed along the edge of the cell membrane in the cytoplasm. Compared to that in the control group, claudin-18 expression showed a significant decrease under hyperoxia. SFTPC expression was reduced on postnatal days 3 and 7 and increased on postnatal days 10 and 14. Podoplanin expression was increased on postnatal day 14. Our preliminary study revealed that transdifferentiation of AECs is abnormally strengthened under hyperoxia. And we found that classic WNT/β-catenin pathway is inhibited under hyperoxia. To investigate the effect of claudin-18 on WNT pathway and AECs transdifferentiation, we transfected claudin-18 overexpression plasmid and found that WNT pathway was activated and AECs transdifferentiation decreased compared with the control group. Therefore, claudin-18 may affect the transdifferentiation of AECs through the WNT pathway, resulting in alveolar dysplasia.

The spatiotemporal expression pattern of claudin-18 during lung development is highly regulated. From embryonic day 16.5 to postnatal day 15, its expression shows an upward trend. Except for postnatal day 3, claudin-18 expression first declines and then reaches a peak at postnatal on day 1 (27). In various animal models of lung injury, decreased expression of claudin-18 has been considered a sign of lung barrier damage (28–31). Especially in a model of hyperoxia-induced BPD, claudin-18 has been shown to possibly be involved in early pulmonary edema development and late alveolar development (32).

Michael et al. (23) studied the role of claudin-18 in lung development and barrier function by producing a claudin-18 (exon 2 and 3) knock-out (KO) mouse model. They found that, compared to wild-type mice, claudin-18 KO mice had lung injury and pulmonary edema, severity of which increased with time, along with an increased alveolar epithelial barrier permeability. When AECs were formed in claudin-18 KO mice, an increase in radial perinuclear aggregates associated with the nuclear plasma membrane was reportedly observed (33). Changes in morphology and function of tight junctions noted in the claudin-18 KO mouse model, namely fewer alveoli, larger in size, widened alveolar compartments, and impaired alveolar formation, were similar to those observed in the rat model of hyperoxia-induced BPD. Human fetal alveoli at a gestational age of 23–24 weeks, which are in the small vesicle stage and still immature, show a state of atelectasis, with widened alveolar interval and increased alveolar fluid exudation. The changes occurred within the risk period of BPD development (34) and may be related to abnormal claudin expression.

Our study showed increased SFTPC expression in neonatal lungs under hyperoxia. Although the transdifferentiation from AEC II to AEC I was thought to be enhanced under these conditions, the expression of SFTPC was decreased in the early stage, which increased later, contrasting the expected trend of transdifferentiation enhancement. The results are consistent with those obtained in previous studies. Hou et al. (35) found SFTPC expression in tissues to first decrease and then increase, while that of the AEC I marker, AQP5, increased in the BPD model. However, primary cells isolated from lung tissues indicated the decreasing trend of SFTPC expression and increasing trend of AQP5 expression. The underlying reason could be that when AEC II transdifferentiation to AEC I increases in tissues, AEC II proliferation is required to maintain the number of cells.

Claudin-18 expression was deemed to be related to peripheral lung epithelial cell maturation (36). Drugs that promote lung maturation could induce claudin-18 expression in human fetal AECs and promote alveolar development, suggesting that claudin-18 may be a potential driver of lung maturation (37). Furthermore, compared to the wild-type group, higher expression of pro-SPC and podoplanin was observed in the claudin-18 KO mouse model (23). Researchers speculated that KO of claudin-18 could have resulted in abnormal AEC differentiation. However, the extent to which claudin-18 expression affects AEC phenotype would need further investigation, and the mechanisms underlying barrier dysfunction and AEC damage remain to be clarified.

In our study, the WNT signalling pathway was inhibited under hyperoxia, possibly enhancing AEC II transdifferentiation. Inhibition of this pathway could reduce cell proliferation, promoting AEC II differentiation to AEC I (38).The expression of β-catenin in embryonic lung respiratory epithelial cells is necessary for the growth and differentiation of peripheral epithelial cell progenitor cells (39, 40). The absence of β-catenin in embryonic lung epithelial cells disrupts lung morphogenesis, restricts the formation and differentiation of peripheral lungs, and enhances the formation of conductive airways (41).

Many recent studies have reported that the WNT signalling pathway is activated by hyperoxia (42–44). Our results differed from the previous ones in at least two aspects. First, we tested five time points after birth, representing a critical period of lung development, when the normal development of the lung is ensured by precise regulation of the WNT pathway. In addition, experiments yielding opposite results were conducted under the intervention of different drugs. Further, despite the BPD model being established in a manner similar to that in previous studies, the feeding conditions and animal batches used were different.

This was the first study that used a neonatal rat model of hyperoxia-induced BPD to investigate the effect of claudin-18 on rat AECs transdifferentiation. And we confirmed that overexpressing claudin-18 could activated WNT and inhibited AECs transdifferentiation. However, a limitation of the study was that we are not sure whether claudin-18 inhibits AECs transdifferentiation by activating canonical WNT pathway, which needs more efforts to explore the molecular mechanisms underlying the obtained results.

## Conclusions

In a neonatal rat model of hyperoxia-induced BPD, changes in claudin-18 and claudin-4 expression were found to follow the same trend as that in the WNT signaling pathway, co-occurring with abnormal AEC transdifferentiation. Therefore, claudin family may be a potential regulator of the WNT canonical pathway, and possibly playing a unique role in AEC transdifferentiation. Nonetheless, this hypothesis warrants further experimental validation.

## Data Availability

The original contributions presented in the study are included in the article/**Supplementary Material**, further inquiries can be directed to the corresponding author/s.
