# Supplementary figures and images for: Claudin-18 expression under hyperoxia in neonatal lungs of bronchopulmonary dysplasia model rats
Source: Front Pediatr. 2022 Oct 10;10:916716. doi: 10.3389/fped.2022.916716 (PMC9589239; doi:10.3389/fped.2022.916716)

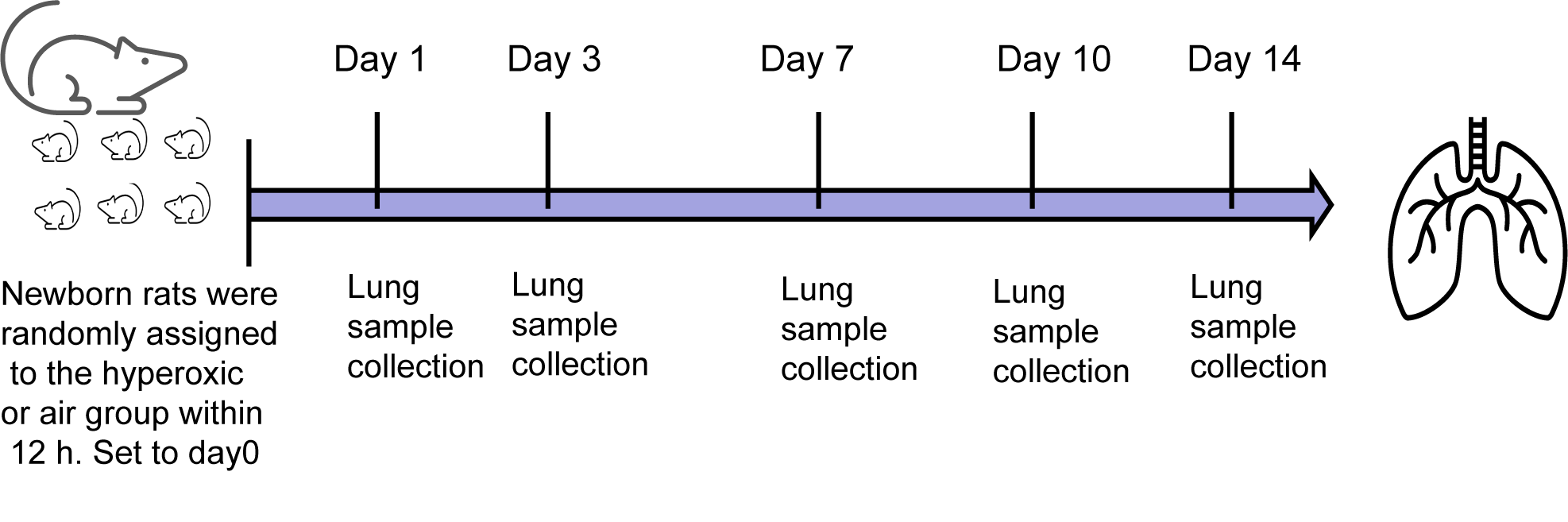

Supplement: Supplementary file 1 [file Image1.tif]

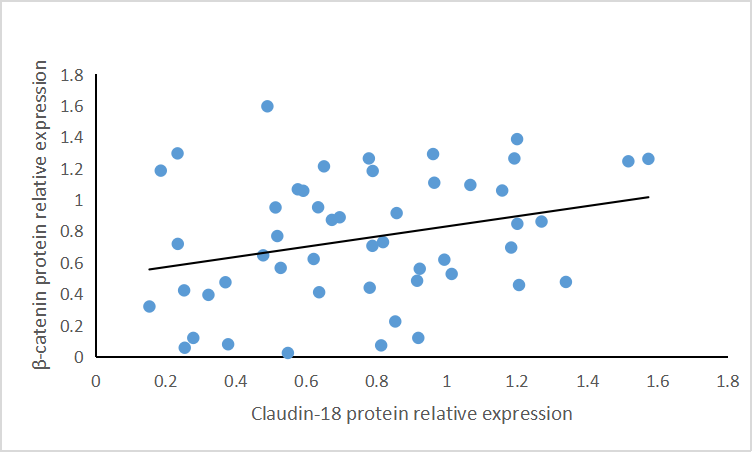

Supplement: Supplementary file 2 [file Image2.tif]
